# A pulmonary rehabilitation program reduces levels of anxiety and depression in COPD patients

**DOI:** 10.1186/2049-6958-8-41

**Published:** 2013-06-22

**Authors:** Athanasios Tselebis, Dionisios Bratis, Argiro Pachi, Georgios Moussas, Ioannis Ilias, Maria Harikiopoulou, Elpida Theodorakopoulou, Silvia Dumitru, Epaminondas Kosmas, Alexandros Vgontzas, Nikolaos Siafakas, Nikolaos Tzanakis

**Affiliations:** 1 Psychiatric Department, “Sotiria” General Hospital of Chest Disease, Athens, Greece; 2 Endocrinology Department, “Elena Venizelou” Hospital, Athens, Greece; 3 Pulmonary Rehabilitation Centre, “Sotiria” General Hospital of Chest Diseases, Athens, Greece; 4 Psychiatric Department, University of Crete, Medical School, Heraklion, Greece; 5 Department of Thoracic Medicine, University of Crete, Medical School, Heraklion, Greece; 6 Department of Social Medicine, Laboratory of Epidemiology, University of Crete, Medical School, Heraklion, Greece

**Keywords:** Anxiety, Depression, COPD, Rehabilitation program

## Abstract

**Background:**

The presence of anxiety and depressive symptoms in COPD patients has been acknowledged for many years. The preponderance of recent studies supports the utility of pulmonary rehabilitation programs to reduce the levels of depression and anxiety in these patients. The aim of this study is to investigate possible changes in levels of anxiety and depression among patients enrolled in a pulmonary rehabilitation program, along with the role of disease severity in these changes.

**Methods:**

In 101 COPD patients, who attended a pulmonary rehabilitation program, levels of trait anxiety (STAI) and depressive symptoms (BDI) were assessed at the beginning and at the end of the program. Age, sex, level of education in years and stage of disease severity were recorded.

**Results:**

Our study included 80 male and 21 female patients. Mean age and mean education level were 64.1 ± 8.1 and 11.3 ± 4.1 years, respectively. Regarding COPD staging, 11 patients suffered from mild, 16 from moderate, 47 from severe and 27 from very severe COPD. Significant decreases in anxiety (from 39.7 to 34.0, p < 0.001) and depression rates (from 10.7 to 6.3, p < 0.001) were observed. A statistically significant reduction in anxiety and depression was revealed (p < 0.05)at all stages of COPD.

**Conclusion:**

Pulmonary rehabilitation programs should be offered to all COPD patients irrespective of disease severity, since they all lead to improvement in anxiety and depressive symptoms.

## Background

Over the last decades, therapeutic evolution in healthcare,in conjunction with an increase in average life expectancy, resulted in a significant and gradual diminution in medical emergencies but simultaneously gave rise to chronic progressive and debilitating diseases.

Gradual reintegration of the chronically suffering patients in family, social and professional environment is nowadays a challenge for the therapeutic team. Social integration is continuously threatened by a constellation of factors concerning the nature of the disease and by the psychosocial parameters implicating the dynamic task of adjustment mechanisms that constitute the subjective experience of the illness.

It is well known that Chronic Obstructive Pulmonary Disease (COPD) is a disease with psychological comorbidities. Several studies have suggested that the prevalence of anxiety and depression among patients with COPD is substantially greater than lifetime rates in the general population and higher than in patients with other pulmonary diseases such as bronchial asthma and tuberculosis [1], or other chronic diseases such as chronic heart failure [2]. International prevalence rates of clinical depression in COPD patients rise above 30% and sometimes above 50% [3], whereas corresponding rates in the background population is around 6-8% [4]. Studies in Greece also report high prevalence rates of depression in COPD patients, often above 42% [1]. Moreover, studies indicate rates of anxiety varying from 10% to 19% [3], which is higher than the prevalence rate of 15% mentioned in the general population [5]. Additionally, one out of two patients with COPD disease appears to suffer from general psychopathological symptoms [6].

Recent studies show a reduction in anxiety and depression symptoms among patients with COPD that are enrolled in pulmonary rehabilitation programs [7-9]; nevertheless some authors believe that further studies are needed [10]. Several studies have pointed out that the degree of psychological improvement brought about by the program depends on disease severity stage. These studies mostly deal with severe COPD (usually leaving out stage I disease) [11,12].

The aim of this study is to investigate the change in anxiety and depressive symptoms among patients with COPD disease who attended a pulmonary rehabilitation program, along with the effect of disease severity on this change. Specifically, we assessed whether the rehabilitation program alters anxiety and depression in patients with COPD, and if this change depends on disease stage, severity and gender.

## Method

### Sample

The study lasted four years and involved all patients with COPD who attended a pulmonary rehabilitation program and met the criteria for inclusion in the study. All interested patients with COPD (for whom there was no contraindication such as angina pectoris, myocardial infarction, severe pulmonary hypertension, congestive heart failure, labile diabetes, inability to exercise due to orthopedic or other reasons, dementia or severe hypoxia caused by exercise and corrected by O_2_ administration) were admitted to this rehabilitation program [13]. The sample consisted of 101 COPD patients, 80 males and 21 females, who completed a three months program. Inclusion criteria were the following: age less than 80 years, without other chronic comorbid medical conditions (cardiovascular diseases, major psychiatric disorders, etc.) and absence of acute exacerbation of COPD during the last two months before starting the program. We excluded patients who did not meet the inclusion criteria (n = 12) as well as those who did not complete the rehabilitation program (n = 43).

### Physical measures

In order to determine COPD severity of our sample, a spirometric evaluation before and after bronchodilation(200 μg salbutamol) was performed. We followed the Global Initiative for Chronic Obstructive Lung Disease (GOLD) diagnostic criteria, which classifies COPD severity [in relation to forced expiratory volume in 1 second (FEV_1_)/forced vital capacity (FVC) ratio (FEV_1_%) - percentage of predicted] into four stages: stage I (mild COPD): FEV_1_ > 80% predicted; stage II (moderate COPD): FEV_1_ 50% to 80% of predicted; stage III (severe COPD): FEV_1_ 30% to 50% of predicted; and stage IV (very severe COPD): FEV_1_ < 30% of predicted [14]. The spirometric evaluation of each patient was performed a few days before he/she started the rehabilitation program.

### Psychological measures

Depression was assessed with the Beck Depression Inventory (BDI) [15], which is widely used, and has been standardized [16] and used in the Greek population previously [17]. The BDI includes 21 items graded from 0 to 3. The inner coherence reliability is high and the re-test reliability ranges from 0.48 to 0.86 for clinical groups and 0.60 to 0.90 for non-clinical population. Its validity in relation to an external criterion for depression, such as clinical diagnosis, is considered to be satisfactory. Anxiety was assessed with the Spielberger State-Trait Anxiety Inventory (SSTAI) [18], one of the well-known and broadly used anxiety rating scales. The inventory consists of 40 items, each one graded from 1 to 4. The SSTAI differentiates anxiety to (a) anxiety caused by a specific condition (state subscale), and (b) anxiety as a more permanent characteristic of the personality (trait subscale). This second (trait) subscale was used in our study protocol. The SSTAI is considered as having a high inner coherence reliability and validity compared to clinical diagnosis. Also it has been standardized [19] and widely used in studies in the Greek population previously [20]. Participants replied to the questionnaires in two phases, at baseline and at discharge from the pulmonary rehabilitation program.

### Pulmonary rehabilitation program

Patients of our study followed a pulmonary rehabilitation program for a period of three months, with three sessions per week, each lasting 50 minutes. The program included respiratory physiotherapy, respiratory muscle training, aerobic exercise on a bicycle ergometer and on a treadmill and strengthening of muscle groups. The exercise was performed with oxygen supplementation while simultaneously recording heart rate and hemoglobin saturation. The minimum and maximum number of sessions per patient was 34 and 39, respectively, with an average of 37 per patient.

### Statistical analysis

The statistical program used was SPSS 16. The statistical analysis was performed using *x*^2^ test, paired *t*-test, ANOVA, sample *t*-test, Pearson correlation and stepwise multiple regression. For regression models, an empirical approach was used after correlation analysis. Statistical significance was set at p < 0.05.

The hospital ethics committee approved the study and all participants provided written informed consent. No financial support was necessary.

## Results

### Sample characteristics

The demographic characteristics of our patients and their spirometric values are summarized in Table 1. There were no differences between genders regarding age, years of education and FEV_1_ percentage of predicted (*t*-test p > 0.05). With regard to the severity of COPD according to GOLD classification, the number of patients with severe COPD disease (47 patients, 46.5% of the total sample) was significantly higher than that of other subgroups (*x*^2^ p < 0.01, Table 1).

**Table 1:** Demographics and baseline characteristics

		**Age**	**Education level**	**FEV**_**1**_	**Depression**	**Anxiety**
			**(years)**	**(% of predicted)**	**(BDI)**	**(STAI)**
**Male**	Mean	64.56	10.90	41.98	10.39	38.88
	N	80	80	80	80	80
	SD	8.06	4.09	21.25	6.62	9.68
**Female**	Mean	62.57	12.67	49.53	11.90	42.62
	N	21	21	21	21	21
	SD	8.38	3.75	22.13	5.72	8.27
**Total**	Mean	64.15	11.27	43.51	10.7	39.65
	N	101	101	101	101	101
	SD	8.13	4.07	21.53	6.45	9.49
Severity according to Global Initiative for Chronic Obstructive Lung Disease (GOLD) diagnostic criteria:						
mild/moderate/severe/very severe**:** 11 (10.9%)/16 (15.8%)/47 (46.5%)/27 (26.7%).						

*BDI* Beck depression inventory, *STAI* Spielberger trait anxiety inventory.

### Changes in anxiety and depression

Mean depression and anxiety scores were significantly lower at discharge compared to baseline for all our patients (paired *t*-test p < 0.05, Table 2). Same findings were revealed for all subgroups (according to staging of COPD disease) of patients and no statistical difference was identified when checking for mean individual changes in depression and anxiety scores among all different disease severity subgroups (ANOVA p > 0.05).

**Table 2:** Means (SD) and differences in BDI and STAI at baseline and after PRP in COPD severity subgroups

**COPD Severity (as per GOLD)**		**Time 1**		**Time 2**		**Difference**	
		**Depression**	**Anxiety**	**Depression**	**Anxiety**	**Depression**	**Anxiety**
		**(BDI)**	**(STAI)**	**(BDI)**	**(STAI)**	**(BDI)**	**(STAI)**
mild	Mean	12.27	43.18	8.36	39.27	−3.91*	−3.91*
N = 11	SD	5.55	8.15	7.21	9.88	5.24	5.86
moderate	Mean	8.44	38.75	4.06	30.62	−4.37*	−8.12**
N = 16	SD	5.66	8.34	2.67	7.59	6.91	8,20
severe	Mean	11.43	39.62	6.85	33.96	−4.57**	−5.66**
N = 47	SD	7.58	10.59	6.04	9.34	5.73	8.08
very severe	Mean	10.15	38.81	5.93	33.93	−4.22**	−4.89**
N = 27	SD	4.75	8.71	5.48	8.39	4.77	5.65
Total	Mean	10.70	39.65	6.33	34.00	−4.38**	−5.65**
N = 101	SD	6.45	9.49	5.69	9.04	5.56	7.30

* Paired *t*-test p < 0.05.

**Paired *t*-test p < 0.01.

*BDI* Beck depression inventory, *STAI* Spielberger trait anxiety inventory.

Mean BDI score, at baseline, for the total of our sample was 10.7, which is significantly (sample *t*-test p < 0.05) higher than the corresponding mean score (5.86) [16] in the general population. At discharge from the program, mean BDI score was 6.33, not statistically different from the corresponding mean score in the general population (sample *t*-test p > 0.05). The rate of patients who had depressive symptoms (BDI > 9) [16] at baseline was 47.5% (48 patients, Table 3) whereas at discharge it became 14.9% (15 patients, Table 3), which was a statistically significant reduction (*x*^2^ p <0.05).

**Table 3:** Severity of COPD and depressive symptoms severity

		**Depressive symptoms severity**								**Total**
		**BDI: 0-9**		**BDI:10-18**		**BDI:19-29**		**BDI > 29**		
		**time one**	**time two**	**time one**	**time two**	**time one**	**time two**	**time one**	**time two**	
COPD Severity (GOLD criteria)	Mild	3	9	7	1	1	1	0	0	11
	Moderate	11	16	4	0	1	0	0	0	16
	Severe	24	36	17	9	5	1	1	1	47
	Very severe	15	25	10	1	2	1	0	0	27
Total		53	86	38	11	9	3	1	1	101

### Changes in anxiety and depression per gender

Regarding anxiety scores our observations indicated that mean scores at baseline were higher than the corresponding scores in the general population. Specifically, female patients of our sample had a mean STAI score of 42.62 (versus 37.47 [19] in the general female population, sample *t*-test p < 0.01), while men had an average of 38.87 (versus 34.54 [19] in the general male population, sample t -test p <0.01). At discharge our study indicated reduced mean STAI scores for both female (36.29) and male patients (33.4). These scores were not statistically different from the corresponding mean scores in the general population (sample *t*-test p > 0.05). The rate of female patients who had clinically significant anxiety symptoms (STAI > 45) at baseline was 47.6% and was reduced to 19% at discharge (*x*^2^ p < 0.05). Regarding male patients of our sample, 25% of them had clinically significant anxiety symptoms (STAI > 43) at baseline and the percentage was reduced to 12.5% at discharge (*x*^2^ p < 0.05).

### Correlations

A significant positive correlation was observed between anxiety and depression scores both at baseline and at discharge from the program (Pearson correlation p < 0.01, Table 4).

**Table 4:** Correlations

**N = 101**		**AGE**	**Education (years)**	**FEV**_**1**_**%**	**BDI 1**	**BDI 2**	**STAI 1**
Education level (in years)	Pearsons’ r	-.063					
	Sig. (2-tailed)	.532					
FEV_1_%	Pearsons’ r	.095	.014				
	Sig. (2-tailed)	.349	.893				
Depression (BDI 1) Time 1	Pearsons’ r	-.076	-.056	.010			
	Sig. (2-tailed)	.447	.575	.923			
Depression (BDI 2) Time 2	Pearsons’ r	-.091	.097	.053	.586**		
	Sig. (2-tailed)	.365	.337	.601	.000		
Anxiety (STAI 1) Time 1	Pearsons’ r	-.223*	-.056	.034	.726**	.408**	
	Sig. (2-tailed)	.025	.575	.741	.000	.000	
Anxiety (STAI 2) Time 2	Pearsons’ r	-.194	.131	.041	.610**	.717**	.691**
	Sig. (2-tailed)	.052	.190	.688	.000	.000	.000

* Correlation is significant at the 0.05 level (2-tailed).

** Correlation is significant at the 0.01 level (2-tailed).

*BDI* Beck depression inventory, *STAI*, Spielberger trait anxiety inventory.

A significant positive correlation was also observed between first and last measuring for both anxiety and depression (Pearson correlation p < 0.01, Table 3), but no correlation was found between FEV_1_% of predicted and anxiety – depression scores (Pearson correlation p > 0.05, Table 4).

More in detail, we implemented stepwise multiple regression analysis using variation in depression as the dependent variable and controlling for gender, age, FEV_1_%, years of education and variation in anxiety (independent variables). Gender, age, years of education and FEV_1_% were not involved in the variance of the dependent variable whereas variation in anxiety interpreted 34.3% of variation in depression (f 1.97 = 50.62, p <0.01). Considering anxiety variation as the dependent variable and gender, age, years of education, FEV_1_% as independent variables the results were not different, since variation in anxiety scores was independent of gender, age and FEV_1_%, whereas variation in depression interpreted 34.3% of variation in anxiety (f 1.97 = 50.62, p <0. 01).

## Discussion

Summarizing the results of this study, we have shown that a rehabilitation program can reduce the high levels of anxiety and depression in patients with COPD. Improvement occurs for patients in all disease stages (with no statistically significant differences among them), irrespective of gender. This improvement is not dependent on disease stage, gender, age or years of education. Spirometry showed no correlation with either anxiety or depression in the course of the program and anxiety and depression were not correlated with the severity of COPD”.

Despite the high prevalence and harmful effects attributed to the comorbidity of anxiety and depression in COPD, only a limited number of studies have addressed their management [21,22].

Drug treatment encounters serious problems. Benzodiazepines may cause respiratory depression and should be avoided [23]. In addition, beta-blockers are contraindicated in these patients, despite their anxiolytic action, because of the potential risk of bronchoconstriction [24]. Atypical antipsychotics in very small doses can alleviate anxiety symptoms in these patients, but they should be used cautiously because of possible neurological and cardiovascular side effects [25]. In other studies SSRIs have been used (first-line drugs for the management of depression) [26-30]. Sertraline [28,29], fluoxetine [26,31] citalopram [32] and paroxetine [30] may improve quality of life, however it is noted that patients with COPD and psychiatric comorbidity are reluctant to take additional medications [26,33].

Both individual and group therapy are useful for the treatment of patients with COPD [34]. The comparison of individual and group intervention usually favors the latter [35-41]. Group therapy is a financially attractive treatment approach that requires few therapists to treat more patients. Furthermore it seems that group therapy offers valuable treatment opportunities, which may be due to recognition of shared experiences and emotions among its members in a situation resembling the real world more accurately [39].

It is very likely that improvement of psychological symptoms in rehabilitation programs is associated with both psychological, and biological parameters (which are closely coupled with the effects of exercise and respiratory physiotherapy).

Biological mechanisms associated with exercise activity, including changes in central monoamine function [36-40], enhanced hypothalamic- pituitary- adrenal axis regulation, increased release of endogenous opioids [42-49] and reduced systemic inflammation [49,50], may affect depression and anxiety among patients undergoing PR. In addition, behavioral mechanisms [51-56] associated with exercise activity as active distraction from worrying thought patterns (rumination), increase in self- efficacy by providing patients with a meaningful mastery experience, provision of daily pleasant events and regular social contact and support, operate synergistically to produce reductions of symptoms.

The fact that patients participate in a pulmonary rehabilitation program, which, with the necessary modifications, works in a way that refers to the functioning of groups formed by people sharing common characteristics [39], acts therapeutically. Moreover, it is well knownthat the sense of belonging to a group is often beneficial, as it provides the opportunity for participants to trigger interactions and through this process to identify elements of personal experience among others, and to process them in a healthier way [57].

The results of this study are consistent with reports of strong evidence of psychological/psychiatric benefits of pulmonary rehabilitation [58-61], (such as improved mood and anxiety) in patients with COPD [62,63]. This study is in accordance with previous findings indicating that patients with less favorable psychological conditions may also benefit from a rehabilitation program [64].

An additional finding is that the effectiveness of a pulmonary rehabilitation program in reducing stress and depressive symptoms experienced by patients with COPD, is undeniable, regardless of disease stage, patients’ gender, age or education level.

Furthermore, this study is in agreement with findings of other related works [29,30] that reported anxiety and depression as being the major comorbidity problem in patients with COPD. However, the prevalence of comorbidity seems to vary widely among different researchers [25,65-67]. The acceptance of common assessment tools for stress and depression in patients with COPD could mitigate the problem.

Finally, the positive correlation between anxiety and depression is a common finding in both the general population [5,20] and in patients with COPD [1,6,68].

The fact that the predicted FEV_1_% and severity of COPD showed no correlation with anxiety or depression has been observed in other studies [6,68]. These observations are consistent with the hypothesis that the predicted FEV_1_% does not reflect all aspects of the disease [69]. It is likely that patients interpret disease seriousness subjectively, which contributes to the development of the levels of anxiety and depressive symptoms.

### Limitations of the study

The purpose of this study was to assess whether a brief three month rehabilitation program can improve levels of anxiety and depression in patients with COPD without being able to answerbut only to speculate on the reasons for this improvement. Subsequent studies should focus on exploring the causes of the improvement.

This was a short-term before-and-after design study (which is most useful in delineating immediate effects of short-term programs). Although it might be more useful in COPD patients to assess longer-term repercussions of the rehabilitation program this was beyond the scope of this study (after all over a longer time period of time conditions may change and obscure any intervention's effects by threatening the study's internal validity). The fact that the number of men was unequal to that of women may actually underestimate baseline anxiety and depression scores, since women tend to have higher levels of both, but such a choice would have eliminated the representativeness of the sample (women with COPD, despite a steady increase in number, still remain fewer than men). An important problem in the study is that a substantial number of patients have chosen to discontinue the rehabilitation program and a subsequent study should examine whether psychological factors are involved in patients attrition. Understanding the problem should not aim at excluding patients with COPD from rehabilitation programs, but to create individualized interventions both before and during rehabilitation.

## Conclusion

Our study supports that pulmonary rehabilitation programs should be offered to all COPD patients regardless of disease severity, since they all get improvements in anxiety and depressive symptoms. Further research would be useful to confirm these findings and to focus on the possibilities of intervention and rehabilitation in patients with mild COPD.

## Competing interests

The authors declare that they have no competing interests.

## Authors’ contributions

AT conceived the experiment, designed the study, performed the psychological measures, collected data, carried out the statistical analysis and drafted the paper; DB performed the psychological measures, carried out the statistical analysis and drafted the paper; AP and GM helped draft the paper; II carried out the statistical analysis and helped draft the paper; EK performed the physical measures and helped draft the paper; MH, ET and SD performed the physical measures; NS and AV supervised the study; NT carried out the statistical analysis, helped draft the paper and supervised the study. All authors read and approved the final manuscript.
